# Platelet-Derived Growth Factor Synergistic Enhancement of Bone Marrow Stimulation for Enhanced Labral Repair and Functional Recovery in a Rat Bankart Model

**DOI:** 10.1177/03635465261465181

**Published:** 2026-07-26

**Authors:** Feabie Mendiaz, Bhavya Vaish, Sumaiya Rifat, Nathan Gray, Le Hoang, Tam Nguyen, Joseph Borrelli, Liping Tang

**Affiliations:** * Department of Bioengineering, University of Texas at Arlington, Arlington, Texas, USA; Investigation performed at the University of Texas at Arlington, Arlington, Texas, USA

**Keywords:** glenoid, labrum, bone marrow stimulation, PDGF, stem cells

## Abstract

**Background:**

Bone marrow stimulation (BMS) is used to treat intra-articular defects, such as articular cartilage defects or to facilitate healing of soft tissue repair; however, BMS yields inconsistent clinical outcomes, potentially due to the inefficient delivery of endogenous progenitor cells.

**Hypothesis:**

Combining BMS with localized placement of platelet-derived growth factor (PDGF) would enhance in situ progenitor cell responses, leading to enhanced labral tissue repair and functional recovery in a rat model.

**Study Design:**

Controlled laboratory study.

**Method:**

A Bankart glenoid labral tear was created in the right shoulder of 50 adult rats. The animals were randomly divided into 5 groups: (1) healthy (baseline), (2) suture only (control), (3) suture + PDGF, (4) suture + BMS, and (5) suture + PDGF + BMS. Healing was assessed at 1.5 (n = 3), 3, and 8 weeks (n = 4). Baseline groups were included at 3 (n = 4) and 8 weeks (n = 5). At each timepoint, labral healing was evaluated by quantifying progenitor cell recruitment, tissue differentiation, and general morphology. Functional recovery was assessed weekly via gait analysis.

**Results:**

BMS alone failed to elicit cell recruitment to the injured labral region. However, the combined BMS + PDGF treatment resulted in a 7-fold increase in progenitor cell accumulation at the tear site compared with BMS alone (27.46 ± 11.51 vs 3.5 ± 3.26 cells/0.01 mm^2^, respectively; *P* < .001). The combined group was characterized by increased chondrogenic differentiation, greater glycosaminoglycan intensity, and significantly lower Pauli score. Furthermore, gait analyses confirmed that the combined treatment yielded the most robust functional recovery.

**Conclusion:**

The synergistic application of BMS + PDGF provided a highly effective method for labral repair compared with isolated treatments, significantly enhancing progenitor cell recruitment, tissue regeneration, and functional recovery.

**Clinical Relevance:**

This study identifies a novel repair technique that overcomes limitations of standard suture-only repair. This strategy offers a promising translational approach to improve outcomes in shoulder labral reconstruction.

The glenoid labrum is a specialized fibrocartilage structure essential for deepening the glenoid fossa and ensuring glenohumeral stability. The glenoid labrum functions by maintaining a “suction cup” effect through negative intra-articular pressure, which significantly increases the glenohumeral contact area.^1,3,20,27^ Labral injuries, often arising from trauma or repetitive stress, compromise the seal, leading to chronic instability and accelerated osteoarthritis.^2,20^ Although glenoid bone loss is a known driver of recurrence, the biological quality of labral healing significantly dictates long-term success. Indeed, incomplete fibrocartilaginous integration, as evidenced by postoperative magnetic resonance imaging, is frequently linked to persistent symptoms and inferior functional outcomes.^2,15,20^

Although arthroscopic suture anchor repair remains the clinical standard for restoring mechanical stability,^11^ the labrum often fails to achieve true histological healing due to its poor intrinsic regeneration/healing capacity, characterized by sparse vascularization and a paucity of endogenous progenitor cells.^1,3,20^ To overcome these regenerative limitations, research has shifted toward biological augmentation. The synovium is a known reservoir of synovial fluid–mesenchymal stem cells (SF-MSCs) capable of contributing to repair.^4,23^ A new strategy using a bioadhesive delivering platelet-derived growth factor (PDGF) as a chemoattractant was found to enhance SF-MSC cell recruitment at the extra-articular repair site. However, this PDGF adhesive-only approach only resulted in incomplete tissue regeneration/healing.^8^ This suboptimal outcome is likely due to the low baseline concentration of SF-MSCs within the synovial fluid,^21^ which provides insufficient cell density for robust regeneration/healing.

Bone marrow stimulation (BMS) has emerged as a strategy to supplement the joint space with a more robust progenitor cell population. By creating controlled subchondral perforations,^47^ BMS allows bone marrow–derived mesenchymal progenitor cells (BM-MSCs) to egress into the joint space^33^ and facilitate fibrocartilage-like repair.^8,27^ Despite the increasing popularity of BMS for treating various synovial injuries, such as chondral defects, its clinical efficacy continues to be a subject of debate (Appendix Table A1, available in the online version of this article).^5,12,16,22,32,33,42,45^ Furthermore, the efficacy of BMS in labral repair remains largely unexamined.^7^ These divergent observations highlight a critical need for controlled studies to evaluate the biological impact of BMS on labral regeneration.

Therefore, we conducted this study using a rat model of Bankart labral tear to evaluate the therapeutic potential of BMS. We sought to address current limitations by testing the hypothesis that combining BMS with a PDGF-releasing bioadhesive would synergistically enhance progenitor cell recruitment and tissue regeneration/healing. We hypothesized that this dual-action approach would lead to potent tissue regeneration and accelerated functional recovery compared with either isolated treatment or standard suture repair.

## Methods

### Study Design

Fifty adult Sprague-Dawley rats (both sexes, 250-350 g) were randomly assigned to 5 groups: (1) healthy baseline, (2) suture-only repair (control), (3) suture + PDGF, (4) suture + BMS, and (5) suture + PDGF + BMS. Repair groups (groups 2-5) were sacrificed at 1.5 weeks (n = 3 per group; total 12), 3 weeks (n = 4 per group; total 16), and 8 weeks (n = 4 per group; total 16). Healthy baseline controls (n = 3 per timepoint) provided benchmarks for 3 and 8 weeks.

The animal study protocol (A2021.0002) was approved by the institutional animal care and use committee at the University of Texas at Arlington under the Animal Welfare Act and the Guide for Care and Use of Laboratory Animals.

### Surgical Procedure

**Figure 1.**
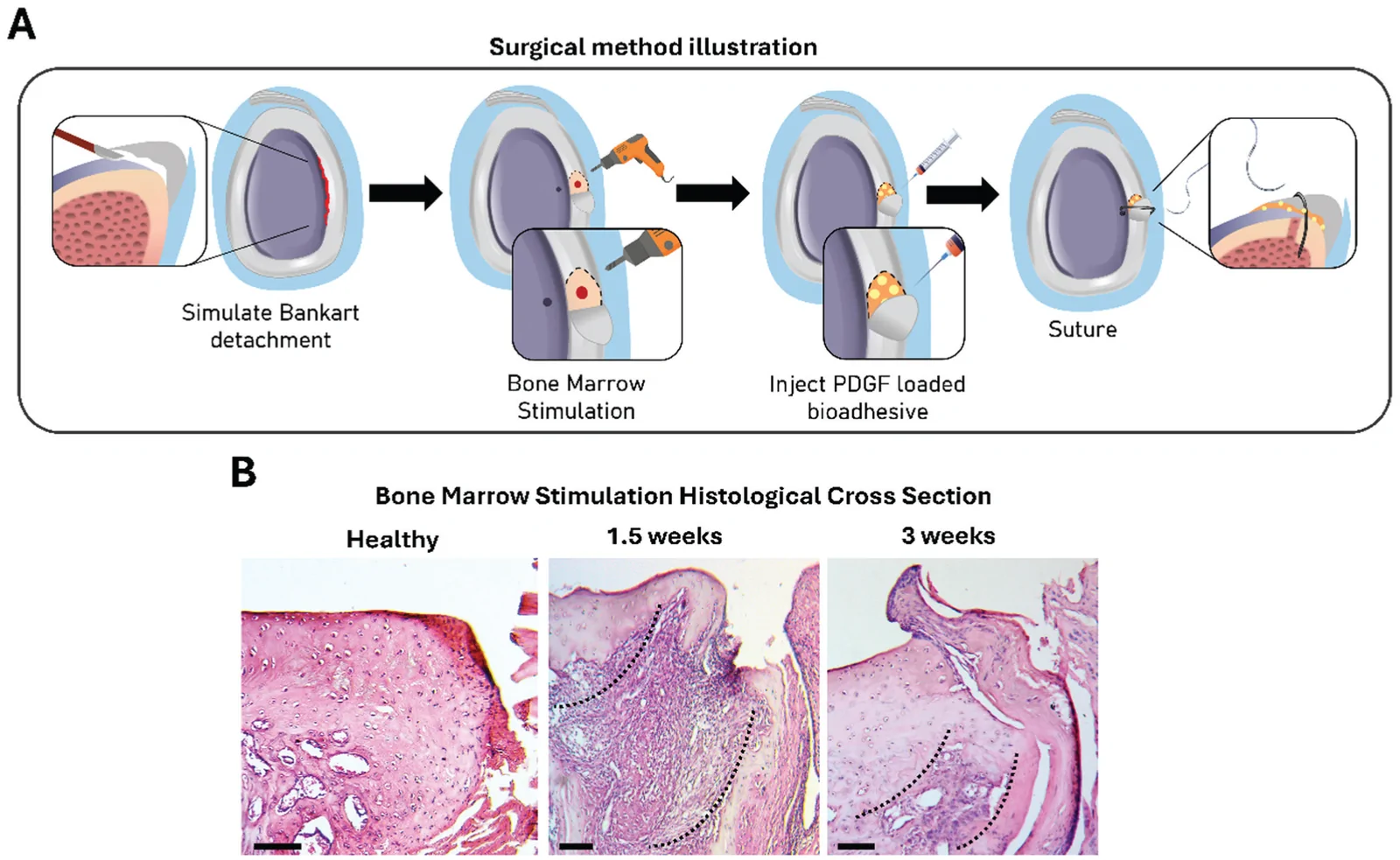
Surgical repair and bone marrow stimulation (BMS) validation. (A) Schematic of the Bankart repair: A 3:00- to 6:00-o’clock detachment was created, followed by sublabral BMS, platelet-derived growth factor (PDGF) adhesive injection, and suture fixation. (B) Representative histological images confirm the BMS channel situated directly beneath the labrum, providing a confirmed route for cell migration from the marrow to the tear. Scale bars = 0.1 mm.

Bankart labral tears were created and repaired using established protocols.^8,9^ Under isoflurane anesthesia, preoperative analgesia (buprenorphine SR), and prophylaxis (cefazolin), a 2- to 3-cm longitudinal incision was made posterolaterally on the right shoulder. After deltoid and rotator cuff division, the shoulder was dislocated to expose the glenoid labrum. A full-thickness detachment (1-mm width) was created from the 3:00- to 6:00-o’clock position. A bone tunnel was drilled through the glenoid ring (0.3-mm bit) for fixation of a No. 5-0 nonabsorbable suture (Prolene polypropylene; Ethicon). In BMS-treated animals, the glenoid rim directly beneath the detached labrum was perforated with the drill bit until visible bleeding confirmed marrow access (Figure 1). For PDGF treatment, a 2-part bioadhesive loaded with 1.20 μg of PDGF-AB (Shenandoah Biotechnology) per 20-µL adhesive/injection site^8,27,28^ was injected into the tear gap using a 31-gauge syringe (Figure 1). The labrum was then secured back to the glenoid rim with the suture knot positioned on the capsular edge to prevent articular interference. To ensure that changes in the labral morphology were attributable to the biological intervention rather than surgical artifact, suture fixation was standardized across all groups. The suture served purely as a mechanical tether; thus, any physical deformation of the tissue at the anchor site was uniform across the study population. After joint relocation and layered closure, animals received daily meloxicam (1 mg/kg) for 3 days.

### Histological Analysis

Explanted shoulder joints (1.5, 3, and 8 weeks) were fixed in 4% paraformaldehyde, decalcified in 10% EDTA for 2 weeks, and paraffin-embedded. After this they were sectioned (7 µm) and stained with hematoxylin and eosin and Safranin O for morphological analysis. Immunohistochemistry was performed using the following primary antibodies to evaluate tissue composition, progenitor cell recruitment, and inflammation: for tissue composition, collagen type 1 (MA1-26771; Invitrogen) and vimentin (MA5-11883; Invitrogen); for progenitor cells, CD44^+^ (60224-1-Ig; Proteintech) and CD73^+^ (12231-1-AP; Proteintech); for bone marrow progenitor cells, CD34^+^ (PA5-85917; Invitrogen); and for inflammation and remodeling, CD11b (PA5-79533; Invitrogen) and MMP-13 (18165-1-AP; Proteintech). Images were captured using a light microscope and quantitatively analyzed using ImageJ software (National Institutes of Health).

### Functional Assessment (Gait Analysis)

Functional recovery was assessed weekly via gait analysis on a custom 24 × 5–inch walkway. We used inkpad prints, digital video, and ImageJ software^9^ to measure and analyze 2 parameters: stride length, which was the distance between consecutive placements of the same forepaw, and step length, which was the distance from forepaw placement strike and the subsequent strike of the opposite forepaw.

### Statistical Analysis

Statistical analysis was conducted using GraphPad Prism Version 8.0.2 (GraphPad Software). A prospective power analysis was performed via G*Power 3.1.9.7 to ensure scientific rigor and adherence to the 3Rs principle of animal welfare.^13^ Based on an alpha of .05 and a power of 0.82, a minimum sample size of n = 3 per group was determined to be sufficient for detecting significant differences in cell recruitment and protein expression. Samples were collected at 1.5 (n = 3), 3, and 8 weeks (n = 4). Baseline groups were included at 3 (n = 4) and 8 weeks (n = 5). Data are reported as mean ± standard deviation. For single timepoint histological data analyses, differences between different groups were compared using 1-way analysis of variance (ANOVA) followed by Tukey post hoc test. Longitudinal gait data were analyzed using a 2-way ANOVA to assess the effect of both treatment group and time, followed by Tukey post hoc test for appropriate multiple comparisons. Statistical significance was defined as *P*≤ .05.

## Results

### Bone Marrow Stimulation Effect on Labral Cellular Responses

**Figure 2.**
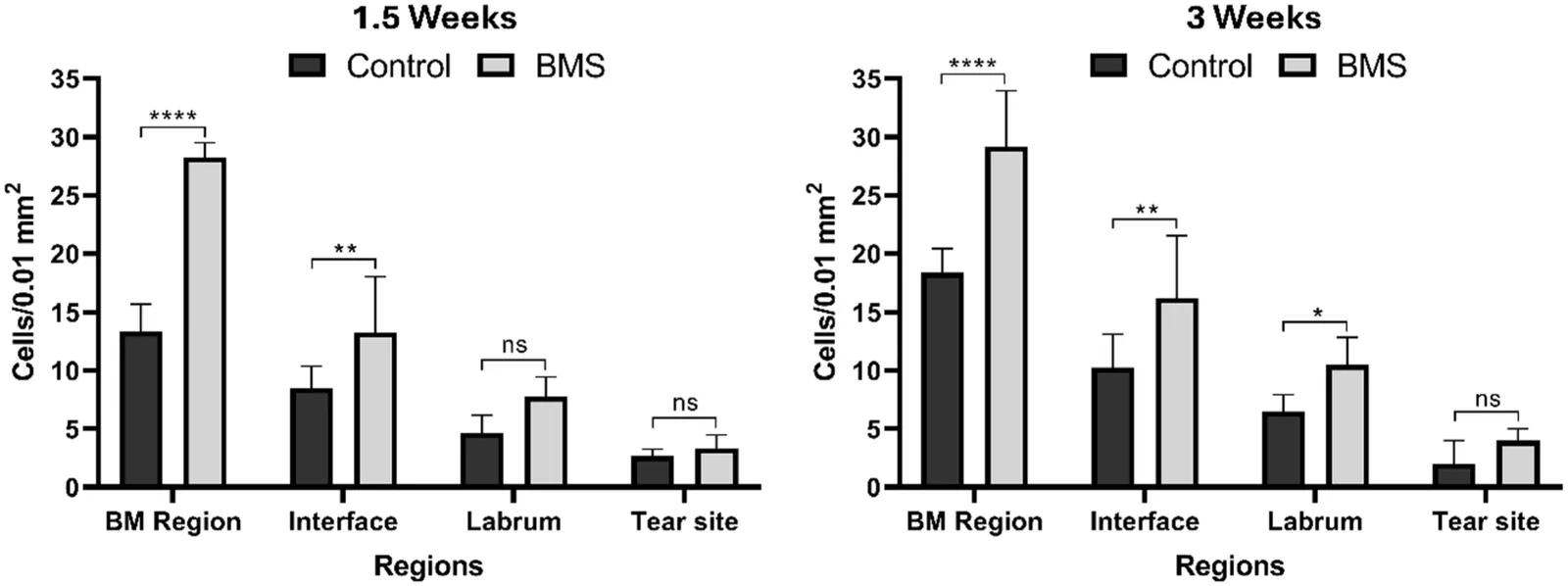
Recruitment of bone marrow (BM)–derived cells to the labral tear site. Bone marrow stimulation (BMS)–induced cell recruitment was quantified by comparing the BMS group to the suture-only (control) group at week 1.5 and week 3. The graph displays the mean migration density of cells recruited from BM regions and along the BM channel leading to the labral tear site. Data presented as mean ± SD (n = 3). Statistical significance was determined via ANOVA with post hoc tests. Significance is denoted by *P < .05, **P < .01, ****P < .0001, and ns (not significant).

To evaluate the effects of BMS on the initial cellular response after labral fixation, we analyzed tissue cellularity after suture-only and BMS treatment. Histological analysis of the suture-only group revealed a significant reduction in overall cellularity within the labral tissue and near the tear site at both week 1.5 and week 3. Conversely, the BMS group showed significantly increased total cell numbers localized to the bone marrow and the bone marrow-labral transition region at both time points compared with suture alone (week 1.5: 28.25 ± 1.26 vs 13.25 ± 4.79 cells/0.01 mm^2^, respectively; *P* < .0001; week 3: 18.38 ± 2.07 vs 10.25 ± 2.87 cells/0.01 mm^2^, respectively; *P* < .0001) (Figure 2). However, neither treatment successfully increased the cell density within the torn labral tissue itself, suggesting that although BMS released cells into the vicinity, they failed to migrate to the injury site.

### BMS Alone Fails to Direct Progenitor Cells to the Tear Site

**Figure 3.**
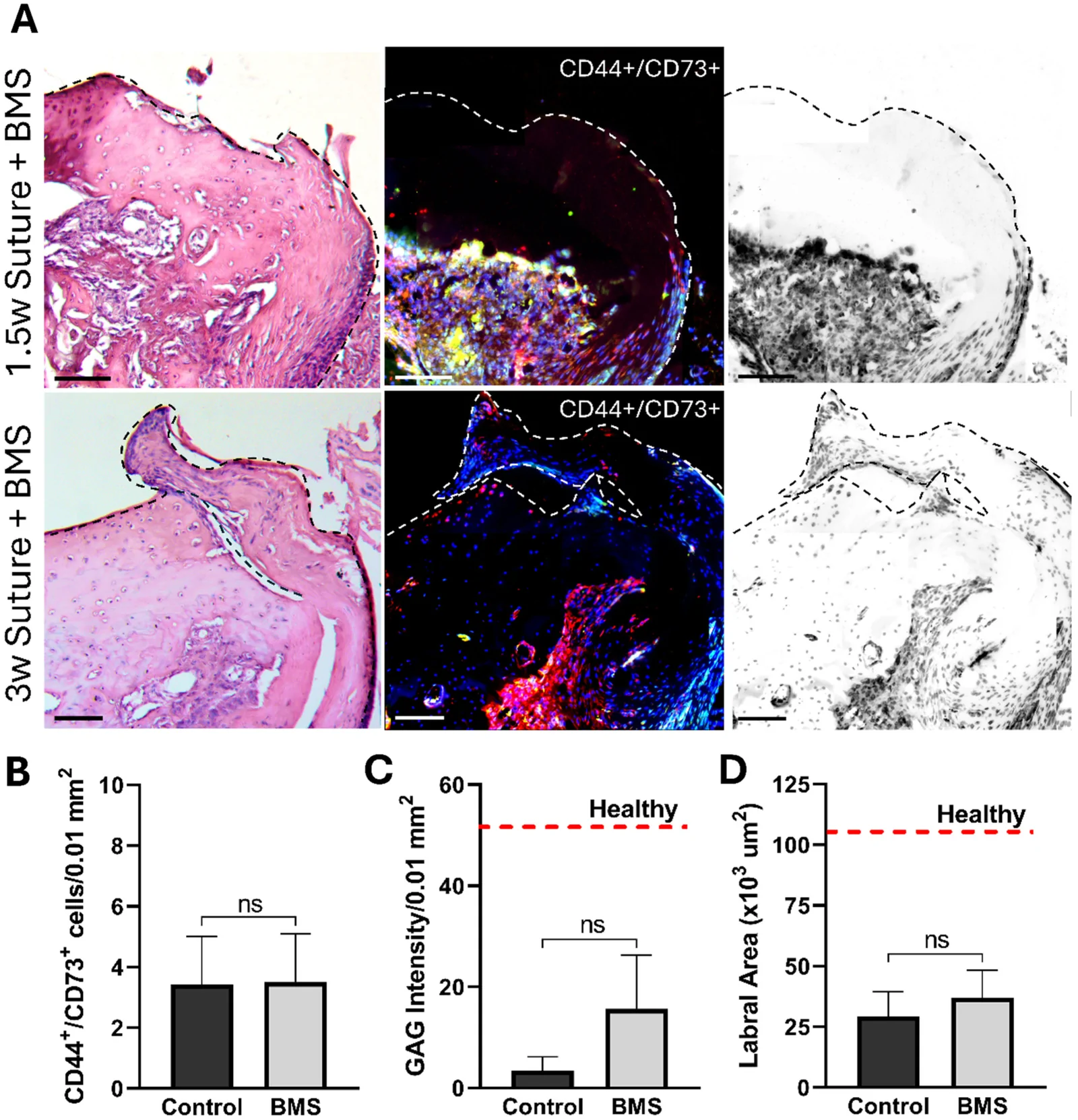
Histological and quantitative assessment of CD44^+^/CD73^+^ progenitor cell recruitment and repair quality. This figure examines the recruitment of progenitor cells and the resulting tissue quality metrics after bone marrow stimulation (BMS) compared with suture-only groups (labeled as “control”). (A) Representative immunohistological images showing the spatial distribution of CD44^+^/CD73^+^ progenitor cells at 1.5 and 3 weeks after BMS (scale bars = 0.1 mm). (B) Quantification of CD44^+^/CD73^+^ cell density at the tear site at week 3. (C) Glycosaminoglycan (GAG) intensity at week 8 as a metric for matrix formation. (D) Total labral repair area at week 8. Data for panels B-D presented as mean ± SD (n = 4). Statistical analysis was performed using ANOVA followed by post hoc tests. ns, not significant.

Further analyses using immunohistochemistry determined the recruitment of CD44^+^/CD73^+^ progenitor cells at the torn labral region. Histological images show that BMS significantly increased CD44^+^/CD73^+^ cell accumulation in and around the bone marrow region (Figure 3A). However, this progenitor cell population failed to reach the tear site at 1.5 or 3 weeks, remaining localized to the bone marrow. Quantification at week 3 showed no statistical difference in progenitor cell count at the tear site between BMS + suture and suture alone (3.50 ± 3.26 vs 3.42 ± 1.60 cells/0.01 mm^2^, respectively; *P* = .94) (Figure 3B). The long-term tissue quality was assessed at week 8 via Safranin O staining. Glycosaminoglycan (GAG) concentration was reduced by ~90% in the suture-only group and ~65% in the BMS group relative to healthy tissue (*P* < .0001 vs healthy) (Figure 3C). Labral area analysis showed strong tissue erosion and poor physical regeneration/healing in both treatment groups, with the healthy labral area being approximately 3-fold greater than either treatment group (Figure 3D). These findings highlight that BMS alone is insufficient for directed migration and adequate extracellular matrix production.

### PDGF Enhances Progenitor Migration and Tear Site Cellularity

**Figure 4.**
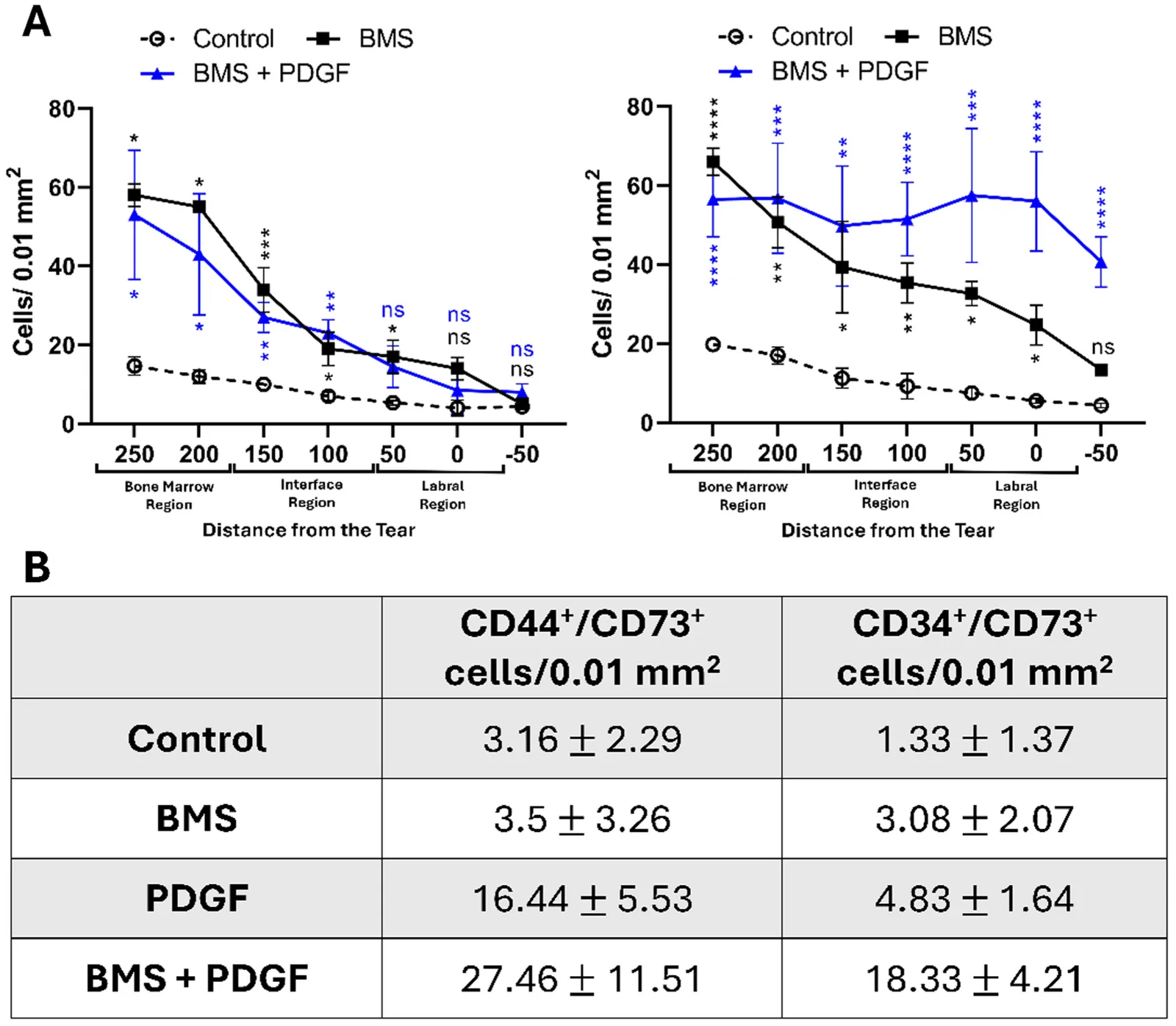
Synergistic impact of bone marrow stimulation (BMS) and platelet-derived growth factor (PDGF)–loaded adhesive on cell migration. To highlight the recruitment dynamics between the bone marrow source and the repair site, 4 primary experimental groups are compared: suture only (control), BMS only, PDGF only, and BMS + PDGF. Results for the additional “healthy native” baseline group used throughout the study are omitted here to focus on repair-site recruitment dynamics. (A) Total migrating cell counts from the bone marrow into the labral tissue at 1.5 weeks (n = 3) and 3 weeks (n = 4). (B) Absolute density and relative percentage of 2 distinct progenitor cell subpopulations: CD44^+^/CD73^+^ and CD34^+^/CD73^+^ cells across the 4 treatment groups at 3 weeks (n = 4). Data presented as mean ± SD. Statistical significance vs control was assessed via ANOVA and Tukey post hoc tests: *P < .05, **P < .01, ***P < .001, ****P < .0001, and ns (not significant). Graph shows cell counts and densities in bone marrow stimulation scenarios with and without platelet-derived growth factor.

The synergistic effects of combining BMS with localized PDGF were evaluated. At week 1.5, both the BMS group and BMS + PDGF group showed similarly low cellularity at the labral region (19.3 ± 5.1 vs 17.6 ± 4.8 cells/0.01 mm^2^, respectively; *P* = .42) (Figure 4A). However, by week 3, the BMS + PDGF group demonstrated significantly elevated cellularity across the bone marrow, transition, and labral regions. Specifically, a 2.1-fold increase in cellularity was determined at the tear site with BMS + PDGF relative to BMS alone (43.7 ± 8.6 vs 20.7 ± 6.2 cells/0.01 mm^2^, respectively; *P* < .001). This temporal lag in maximal accumulation aligns with physiological stages of growth factor–mediated recruitment and local proliferation. These findings show that combined BMS and PDGF successfully directed progenitor cell migration from bone marrow to the injury site.

### Progenitor Cell Source Quantification

Progenitor cell origins at week 3 were assessed using 2 marker sets: CD44^+^/CD73^+^ (general progenitor/synovial-derived) and CD34^+^/CD73^+^ (bone marrow-derived). Although PDGF alone increased CD44^+^/CD73^+^ cell numbers, it did not significantly increase marrow-derived CD34^+^/CD73^+^ cells (Figure 4B). However, the BMS + PDGF significantly increased both CD44^+^/CD73^+^ and CD34^+^/CD73^+^ progenitor populations compared with BMS alone (total: 27.46 ± 11.51 vs 3.5 ± 3.26 cells/mm^2^, respectively; *P* < .001) (Figure 4B). Ratio analysis (CD34^+^/CD73^+^ cell density divided by CD44^+^/CD73^+^ cell density) revealed that a majority (66.8%, derived from 18.33 bone marrow–derived cells out of 27.46 total cells/mm^2^) of the recruited progenitors in the BMS + PDGF group were bone marrow derived. These results confirmed that the BMS + PDGF treatment triggered high recruitment of progenitor cells from both synovial and bone marrow sources.

### Enhanced Tissue Regeneration at Week 8

**Figure 5.**
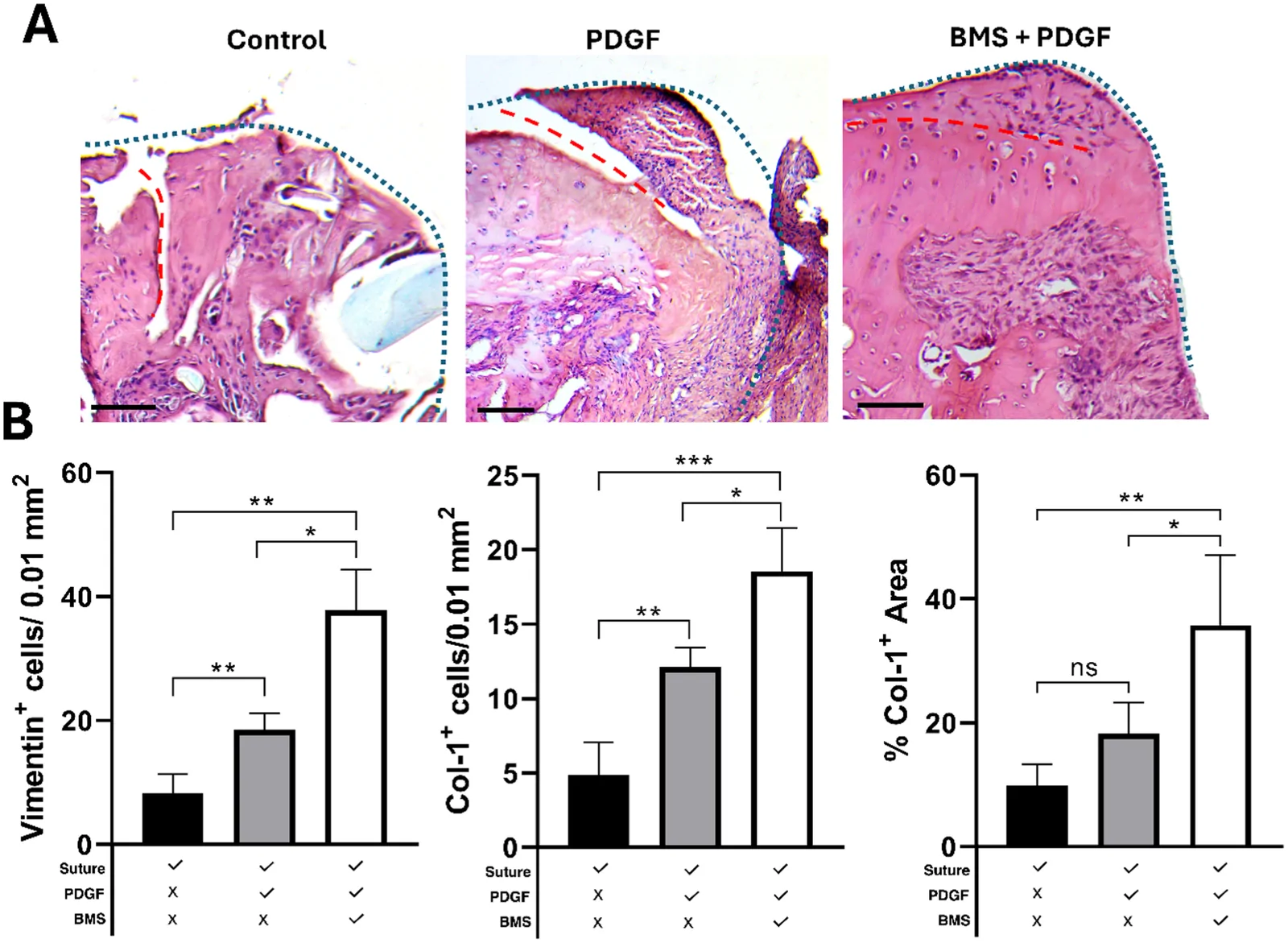
Regenerative tissue responses and matrix composition at week 8. Evaluation of regenerative capacity for suture only (control), bone marrow stimulation (BMS), and BMS + platelet-derived growth factor (PDGF) combination. (A) Representative histological images showing labral morphology and tissue integration of the repaired labrum (scale bar = 0.1 mm). (B) Quantification analysis of the tear site including vimentin^+^mesenchymal cell density, collagen I^+^ fibroblast density, and the percentage of collagen I^+^ labral area. Data presented as mean ± SD (n = 4). A 1-way ANOVA followed by post hoc testing was performed for statistical analysis: *P < .05, **P < .01, and ***P < .001.

Increased progenitor cell responses led to enhanced labral tissue regeneration/healing and matrix formation at week 8. Expression of vimentin (hereafter designated as vimentin^+^ cells), a marker associated with chondrogenesis and structural support,^7,10,44^ was significantly higher in the BMS + PDGF group compared with other groups (BMS + PDGF 37.67 ± 7.03, PDGF 19.67 ± 3.20, and suture 9.67 ± 1.41 cells/0.01 mm^2^; *P* < .05) (Figure 5B). Although PDGF treatment alone significantly increased collagen I postive (or collagen I^+^) cell recruitment and production, consistent with previous findings,^8^ the BMS + PDGF produced the highest collagen I^+^ cell recruitment and subsequent production among all treatment groups (Figure 5B). These findings support that the increased progenitor cell recruitment and differentiation promoted by the BMS + PDGF synergy resulted in significant extracellular matrix production in the torn labral regions.

### Enhanced Tissue Morphology and Quality

**Figure 6.**
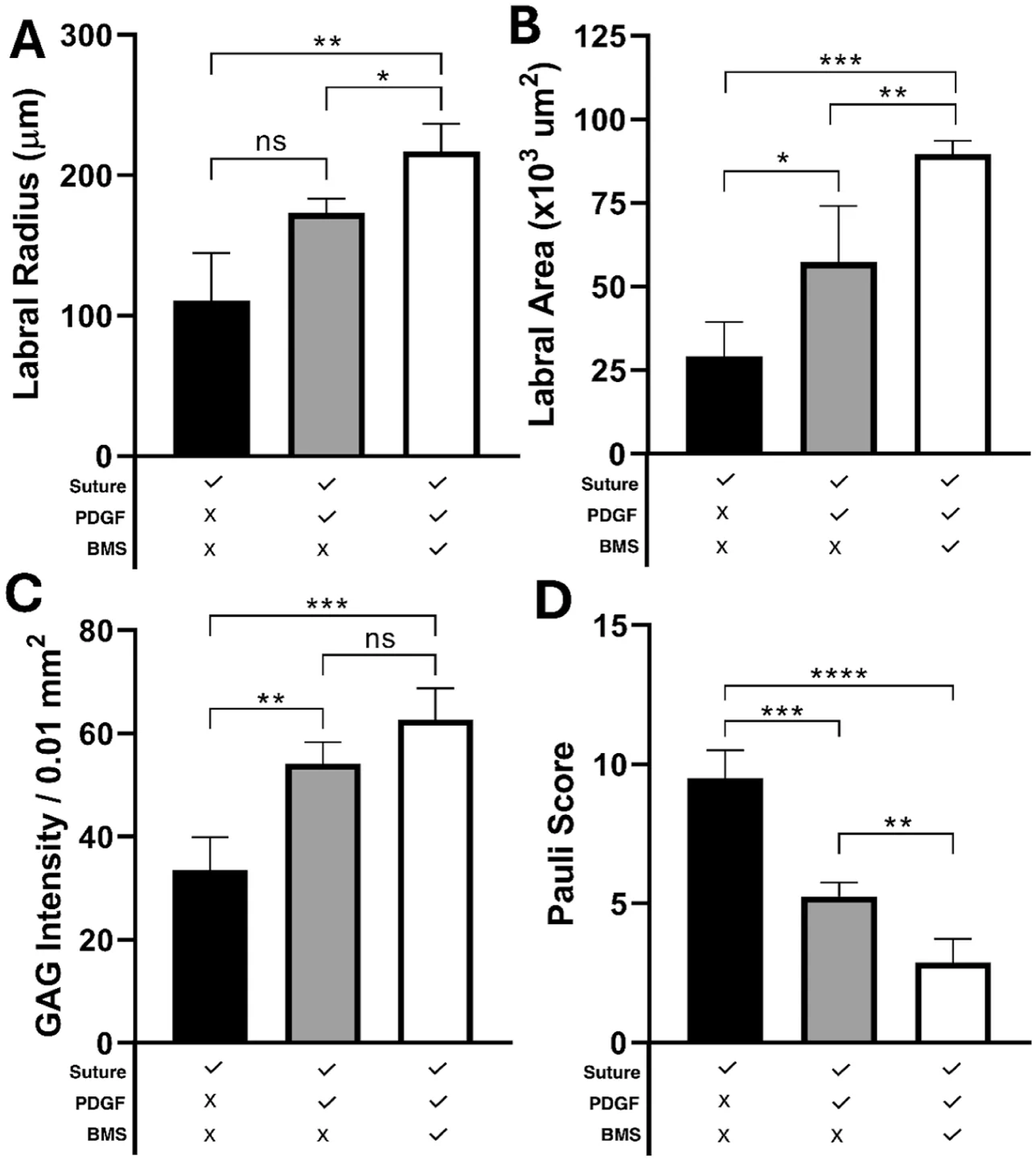
Long-term labral tissue quality and morphological recovery. Comprehensive comparison of suture only (control), platelet-derived growth factor (PDGF), and bone marrow stimulation (BMS) + PDGF at week 8. The regenerative outcomes are quantified by (A) labral radius, (B) labral area, (C) glycosaminoglycan (GAG) intensity, and (D) Pauli score (overall tissue quality). Data presented as mean ± SD (n = 4). A 1-way ANOVA followed by post hoc testing was performed for statistical analysis: *P < .05, **P < .01, ***P < .001, ****P < .0001, and ns (not significant).

To determine the overall tissue regeneration, we quantified and compared the labral tissue dimensions (including radius and cross-sectional surface area), tissue GAG intensity, and Pauli scores among different treatment groups at 8 weeks posttreatment.^37^ Among all treatment groups, the suture-only (control) group exhibited the smallest labral radius of curvature at 8 weeks, reaching only 44.97% ± 17.77% of healthy levels (Figure 6A). Although PDGF-only treatment provided a modest improvement over suture-only repair, reaching 68.95% ± 8.39% of healthy levels, the BMS + PDGF treatment produced the highest degree of tissue regeneration, with a labral radius of curvature (84.45% ± 10.44%) closest to healthy levels (Figure 6A). Trends in labral area measurements were consistent with the radius of curvature measurements. Specifically, the treatment effect on labral area reduction followed this order: BMS + PDGF (19.53% ± 5.29%) < PDGF (41.61% ± 19.26%) < control (69.68% ± 13.56%) (Figure 6B). GAGs are major components of extracellular matrix in the labrum.^31^ Subsequent tissue analyses were performed using Safranin O staining to assess GAG content in the labral tissue at 8 weeks. In agreement with the morphological findings, the effect of treatments on GAG production followed this order: BMS + PDGF (62.71 ± 6.03 intensity/0.01 mm^2^) > PDGF-only (54.15 ± 4.17 intensity/0.01 mm^2^) > control (33.54 ± 6.38 intensity/0.01 mm^2^) (Figure 6C). Finally, the quality of labral fibrocartilage tissue was assessed using the Pauli grading system.^37^ With healthy tissue graded as 1, we found that BMS + PDGF combined treatment produced the lowest (best) score (2.80 ± 0.84), the suture-only treatment yielded the highest (worst) score (9.5 ± 1.0), and the PDGF-only treatment produced a score of 5.25 ± 0.50 (Figure 6D).

### Functional Consequences of Combined Treatment

**Figure 7.**
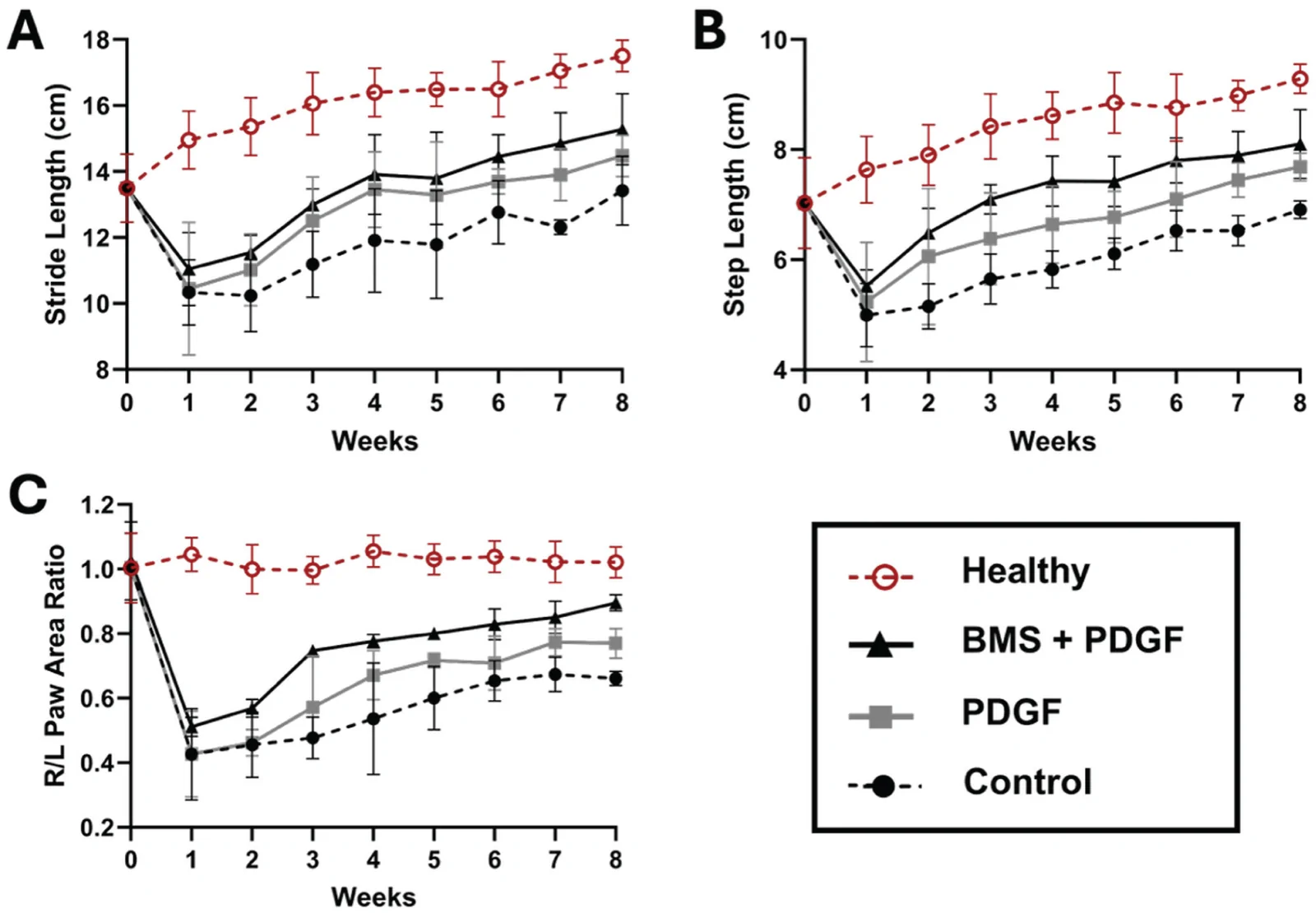
Longitudinal assessment of functional shoulder recovery via gait analysis. The recovery of shoulder function over time was monitored weekly over 8 weeks across suture only (control), platelet-derived growth factor (PDGF) adhesive, and bone marrow stimulation (BMS) + PDGF groups, with healthy animals as a baseline. Parameters include (A) stride length, (B) step length, and (C) width/paw area. Data presented as mean ± SD (n = 3 per group).

To evaluate the long-term functional consequences of the differential healing observed histologically, gait analysis was performed weekly over the 8-week postoperative duration using healthy rats as a baseline. Suture-only (control) rats exhibited a significant and sustained functional deficit throughout the 8 weeks, showing negligible recovery in key gait parameters: ipsilateral to contralateral step length (*P* < .001), stride length (*P* < .001), and paw area ratio (*P* < .001). Treatment with PDGF alone demonstrated consistent but modest improvements across all gait parameters, indicating a limited positive effect on recovery. The combined BMS + PDGF treatment resulted in the most substantial functional recovery, successfully restoring gait parameters to approximately 85% of healthy baseline values by week 8. Importantly, when compared with suture-only control rats at the 8-week endpoint, the combined BMS + PDGF treatment group demonstrated statistically significant improvements across all functional parameters: step length (*P* = .0069), stride length (*P* = .0242), and paw area ratio (*P* = .0474) (Figure 7). These findings establish a direct correlation between the biologically robust tissue regeneration, characterized by the highest progenitor cell recruitment, GAG intensity, and low Pauli score, and the substantial functional restoration of the shoulder. Overall, these findings highlight the unique therapeutic efficacy of the BMS + PDGF synergy.

## Discussion

BMS techniques have long been the gold standard for facilitating biological repair in arthroscopic joint surgery, largely due to their surgical simplicity and minimal cost.^12,33,46^ The underlying premise is the creation of a physical conduit between the intra-articular space and the subchondral bone marrow—a reservoir rich in mesenchymal stem cells (MSCs) and progenitor populations.^25^ The theorized repair cascade involves MSC recruitment, attachment, “nesting” within the lesion, and subsequent proliferation and matrix production.^17,36^ However, our results challenge the adequacy of BMS as a standalone therapy for labral tears. Although the positioning of BMS drill holes is a critical surgical variable, the results suggest that proximity alone may not suffice for optimal repair. We observed that even with a successful cell release, cells remained largely sequestered at the drill hole sites without effectively repopulating the torn labrum. Furthermore, placing BMS sites directly within the injured footprint carries the risk of compromising the structural integrity of the subchondral bone, which already may be weakened by inflammatory bone resorption after the initial tear. This spatial mismatch and potential for structural compromise likely contribute to “low-quality” repair often characterized by fibrocartilage erosion and eventual mechanical failure.^16^ These findings align well with increasing clinical evidence suggesting that although BMS can initiate a healing response, the resulting tissue often lacks the structural longevity required for durable joint stabilization.^5,12,16,22,32,33,42,45^

A critical observation in our study was the temporal shift in cellular density. We noted a distinct lack of cellularity at the 1.5-week interval, followed by a significant peak at 3 weeks. This lag is highly representative of the physiological stages of growth factor–mediated recruitment.^39^ In a chronic or posttraumatic injury state, the labral tissue, as fibrocartilage with naturally low cell density, often becomes “chemotactically silent,” failing to express the endogenous ligands necessary to direct cell migration across the joint interface.^6,20^ The 3-week peak observed in our BMS + PDGF group reflects the time required for progenitor cells to sense the exogenous PDGF gradient, navigate the fibrin scaffold, and undergo local expansion at the labrum-to-bone interface. This finding is consistent with established models of marrow-stimulated healing,^32,42,45^ where early molecular signaling must precede visible histological cell infiltration. By supplementing the site with a sustained PDGF signal, we effectively “primed” the injury environment, ensuring that the initial burst of cells that were released by BMS did not dissipate into the synovial fluid but were instead actively recruited to the repair site.

**Figure 8.**
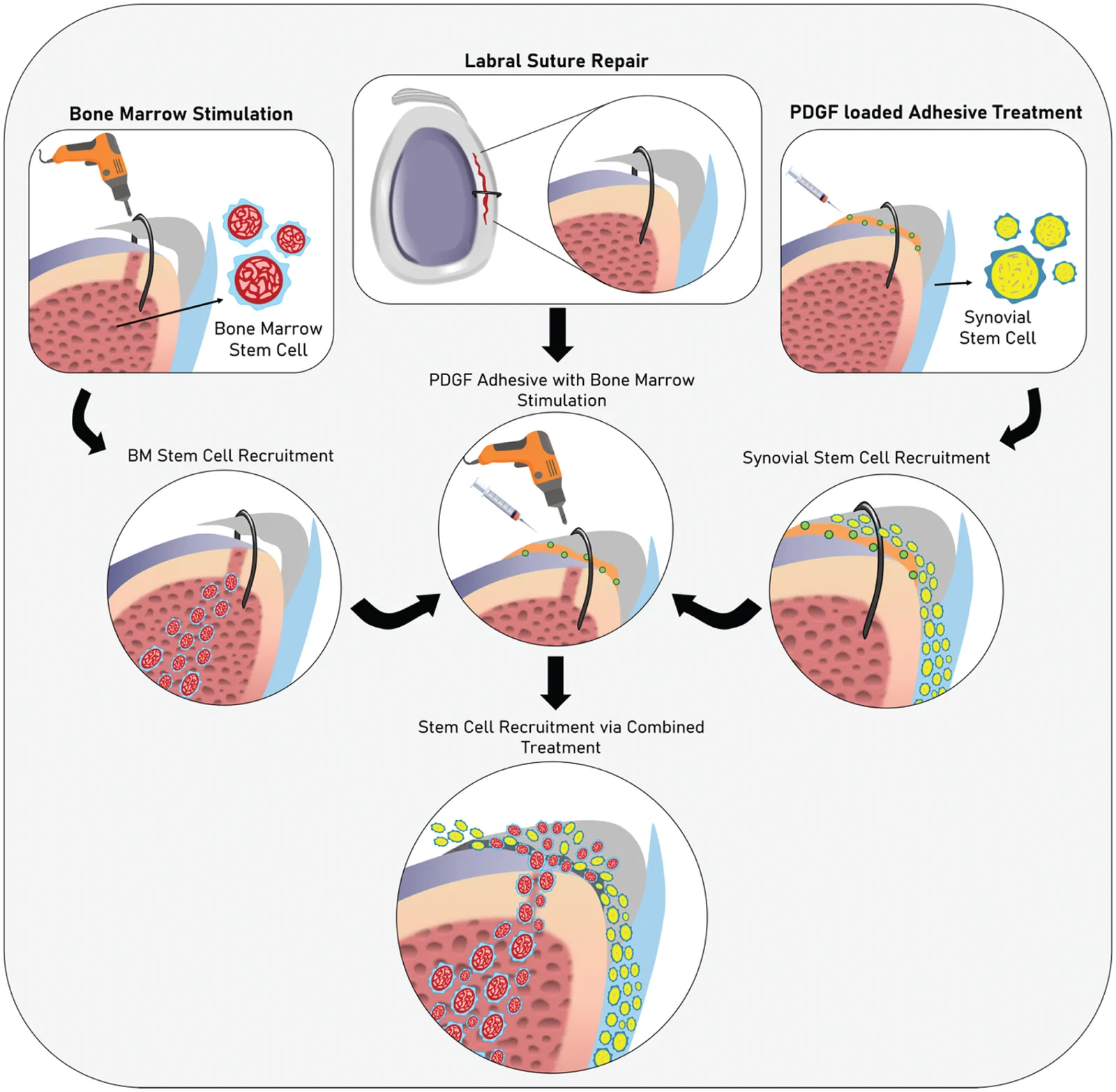
Schematic summarizing the enhanced, progenitor cell-mediated mechanism of combined bone marrow stimulation (BMS) and platelet-derived growth factor (PDGF) treatment for labral regeneration. This schematic illustrates the proposed mechanism by which the combined treatment (BMS + PDGF) synergistically enhances the labral regenerative responses after an in vivo Bankart-like labral tear. BMS alone provides only limited progenitor cell recruitment due to the absence of a sufficiently strong, sustained chemotactic signal at the tear site. This limitation is overcome by supplementing the treatment with the PDGF-loaded adhesive applied directly at the labral tear site, providing a sustained, powerful chemotactic gradient. The combined treatment of BMS (source) and PDGF (signal) is expected to significantly increase the recruitment and proliferative response of progenitor cells from the bone marrow to the labral tear. This enhanced progenitor cell–mediated regenerative response ultimately leads to significantly improved labral healing outcomes, including improved labral morphology, better overall extracellular matrix composition (glycosaminoglycan and collagen type 1 content), and enhanced long-term shoulder functional recovery.

A primary limitation of traditional BMS is the absence of chemotactic signals to guide cell migration and retention. Although a fibrin clot may form a temporary bridge,^43,47^ it is often insufficient to facilitate MSC migration to the tear site without bioactive augmentation. Our results demonstrate a 7-fold increase in progenitor recruitment when BMS was paired with PDGF, suggesting a powerful synergistic mechanism. PDGF functions here in 2 distinct capacities. First, it acts as a potent mitogen and chemoattractant, driving the “homing” of MSCs toward the PDGF-loaded adhesive.^8,27^ Second, the bioadhesive itself provides a stable structural interface that supports cell attachment and retention—a critical requirement in the dynamic,^8,27^ fluid-filled environment of the shoulder joint.^8,36^ Beyond mere recruitment, our data indicate that PDGF plays a role in phenotypic commitment of recruited cells. The biochemical signaling provided by PDGF likely helps steer the recruited progenitor population toward a functional fibrochondrogenic lineage,^6,39^ preventing the random differentiation often seen in unguided marrow stimulation. This proposed mechanism is summarized in Figure 8.

A common criticism of BMS is that it leads to “fibrotic” repair tissue rather than “hyaline-like” cartilage.^40^ However, it is essential to consider the unique anatomy of the glenoid labrum. Unlike the weightbearing hyaline cartilage of the humeral head, the labrum is inherently a dense, fibrocartilage structure designed to resist tension and enhance joint depth.^19,20^ Our quantitative analysis showed that the BMS + PDGF treatment produced a repair tissue rich in GAG and collagen type 1, which closely mimics the native transition zone.^38^ Notably, the morphological changes in the control group were characterized by a global alteration in the radius of curvature throughout the repair site, suggestive of tissue atrophy or necrotic degradation rather than localized mechanical tension. Although the control and BMS-only tissue showed significant tissue atrophy, the structural preservation seen in the combined treatment group reinforces the hypothesis that labral “regeneration” does not necessarily require hyaline cartilage but rather a highly organized, dense fibrocartilage. The lack of significant curvature improvement in the PDGF-only group, despite identical suture tension, reinforces the hypothesis that structural preservation depends on the synergistic effect of BMS-mediated cell recruitment and growth factor signaling.

It is worth noting that the BMS procedure does not act in a vacuum. The creation of a marrow-derived fibrin clot releases a cocktail of endogenous factors, including fibroblast growth factor, vascular endothelial growth factor, insulin-like growth factor 1, transforming growth factor β, and platelet-derived growth factor AB,^29,34,50^ which support angiogenesis and chondrogenesis.^41,48,49^ However, our results suggest that these endogenous signals are often too transient or too weak to achieve total labral regeneration.

Our exogenous PDGF signal appeared to act as the dominant directive, organizing the background noise of the endogenous environment into a focused regenerative response. Although the 3-week interval was marked by peak progenitor cell recruitment, the 8-week cohort demonstrated a transition toward functional tissue synthesis. At this later point, the cellular profile shifted from undifferentiated proliferation to a mature fibrochondrogenic phenotype.^6,38^ A sustained increase in vimentin^+^ cell density suggests that the early cellular influx successfully differentiated into the functional units required for the observed improvements in structural tissue quality and matrix organization.^7,10,44^ This maturation process is essential for clinical success, as it marks the transition from a temporary cellular influx to a permanent, load-bearing tissue.

From a clinical perspective, the integration of PDGF with BMS offers a promising pathway for augmenting current surgical techniques, such as suture anchor repair for Bankart lesions.^30^ Rather than relying on a static protein coating, the use of PDGF-loaded hydrogels or adhesives allows for controlled, sustained diffusion. This ensures that a chemotactic gradient is maintained throughout the critical 3-week recruitment window identified in our study.

The translation of PDGF to labral repair is supported by its established clinical use as a localized chemoattractant in other orthopaedic procedures. Specifically, its safety and efficacy have been demonstrated in the FDA-approved treatment of chronic diabetic foot ulcers and the regeneration of alveolar bone after periodontal infection.^14^ Similar recruitment of tenocytes and fibroblasts has been observed in rotator cuff repair models,^18^ suggesting that PDGF can successfully bridge the biological gap at the fibrocartilaginous interface—a key challenge in augmenting labral repairs. Applying these principles to shoulder orthopaedics could address the vascular and cellular deficiencies that often lead to high failure rates in chronic labral repair.^20^

Although these results are encouraging, they must be interpreted within the constraints of the rat model. The relatively small size of the rat glenoid means that even a minor surgical deviation can significantly affect the healing environment. Nevertheless, statistical power for these comparisons was maintained using a multigroup ANOVA (E = 15-16), which is robust for detecting significant morphological shifts in this established rat model. Our use of a rigid drill bit for subchondral perforation may have introduced thermal necrosis, potentially underestimating the regenerative potential that could be achieved with microfracture awl or newer subchondroplasty technique.^24,35^ Furthermore, although our gait analysis provides a sensitive functional indicator of recovery,^26^ it is important to recognize that such measures are influenced by both pain management and structural healing. The significant improvements in gait parameters observed in the BMS + PDGF group likely reflect a synergistic outcome where biological regeneration reduces the mechanical and inflammatory stimuli for pain. By using a standardized postoperative analgesic protocol to minimize acute discomfort, we ensured that the observed gait improvements served as a functional indicator of the ongoing tissue regeneration process. As healing progresses and the labral structural integrity is restored, there is a characteristic reduction in protective gait patterns, signaling a return toward native joint kinematics.^26^ However, future studies are needed to prioritize direct biomechanical testing. Quantifying the stiffness, tensile strength, and pullout resistance of the regenerated labral tissue will be essential for validating these findings before moving into larger animal models or human clinical trials. Such data will confirm whether the morphological and histological improvements we observed translate into durable, long-term joint stability under physiological loading.

Although the positioning of BMS drill holes is a critical surgical variable, the results of this study suggest that proximity alone may not suffice for optimal repair. Placing BMS sites directly within the injured footprint carries the risk of compromising the structural integrity of the subchondral bone, which may already be weakened by inflammatory bone resorption after the initial tear. Furthermore, our results indicate that the benefit of PDGF extends beyond mere recruitment; it plays a significant role in the phenotypic commitment of recruited cells. This suggests that the biochemical signaling provided by PDGF is likely necessary to transform the recruited progenitor population into the functional fibrochondrocytes required for true labral regeneration.

## Conclusion

This study demonstrated that the synergistic application of BMS and PDGF provided an advanced method for labral repair compared with isolated treatments. By drastically enhancing progenitor cell recruitment, this combined approach significantly overcame the spatial and migratory limitations inherent to standalone BMS. The resulting cellular influx translated directly into enhanced tissue architecture, restoration of native curvature, and measurable functional recovery. These findings underscore the clinical promise of integrating targeted chemotactic signaling (PDGF) with an endogenous progenitor cell source (BMS) to bridge the “biological gap” in routine, suture-only repairs. Continued refinement of this autologous-driven strategy in larger models will be essential to validate long-term durability, potentially establishing a more reliable standard of care for complex fibrocartilaginous injuries in patients.

## Supplemental Material

sj-docx-1-ajs-10.1177_03635465261465181 – Supplemental material for Platelet-Derived Growth Factor Synergistic Enhancement of Bone Marrow Stimulation for Enhanced Labral Repair and Functional Recovery in a Rat Bankart Model Supplemental material, sj-docx-1-ajs-10.1177_03635465261465181 for Platelet-Derived Growth Factor Synergistic Enhancement of Bone Marrow Stimulation for Enhanced Labral Repair and Functional Recovery in a Rat Bankart Model by Feabie Mendiaz, Bhavya Vaish, Sumaiya Rifat, Nathan Gray, Le Hoang, Tam Nguyen, Joseph Borrelli and Liping Tang in The American Journal of Sports Medicine
