# Supplementary material for: Platelet-Derived Growth Factor Synergistic Enhancement of Bone Marrow Stimulation for Enhanced Labral Repair and Functional Recovery in a Rat Bankart Model
Source: Am J Sports Med. 2026 Jul 26;54(11):2740–52. doi: 10.1177/03635465261465181 (PMC13530368; doi:10.1177/03635465261465181)
Supplement: Supplementary file 1 [file sj-docx-1-ajs-10.1177_03635465261465181.docx]

Short-term clinical outcomes (< 5 years) are mixed,^6–9^ and long-term data (> 5 years) raise concerns regarding mechanical inferior repair tissue and high rates of conversion to arthroplasty.^2,10^

**Table S1:** Short term (<5 years) and long term (>5 years) clinical outcomes after the bone marrow stimulation treatment in a variety of clinical scenarios. ^1–6,9,10^ Data represents mean follow up time, description of the treatment, sample size, and key outcomes from the studies.

**References**

1.	Benjamin Domb LG, Gupta A, Dunne KF, et al. Microfracture in the Hip: Results of a Matched-Cohort Controlled Study With 2-Year Follow-up Microfracture in the Hip Results of a Matched-Cohort Controlled Study With 2-Year Follow-up. *Am J Sports Med Downloaded from by BENJAMIN DOMB on*. 2015;43.

2.	Domb BG, Rybalko D, Mu B, Litrenta J, Chen AW, Perets I. Acetabular microfracture in hip arthroscopy: clinical outcomes with minimum 5-year follow-up. *HIP International*. 2018;28(6):649-656.

3.	Gobbi A, Karnatzikos G, Kumar A. Long-term results after microfracture treatment for full-thickness knee chondral lesions in athletes. *Knee Surg Sports Traumatol Arthrosc*. 2014;22(9):1986-1996.

4.	Kahana-Rojkind AH, Rodriguez JD, Sikligar D, McCarroll TR, Quesada-Jimenez R, Domb BG. Enhancing Labral Healing in Arthroscopic Hip Surgery: The Marrow Venting Technique. *Arthrosc Tech*. Published online December 12, 2024:103347.

5.	Migliorini F, Simeone F, Bardazzi T, et al. Regenerative Cartilage Treatment for Focal Chondral Defects in the Knee: Focus on Marrow-Stimulating and Cell-Based Scaffold Approaches. *Cells*. 2025;14(15).

6.	Millett PJ, Huffard BH, Horan MP, Hawkins RJ, Steadman JR. Outcomes of Full-Thickness Articular Cartilage Injuries of the Shoulder Treated With Microfracture. *Arthroscopy - Journal of Arthroscopic and Related Surgery*. 2009;25(8):856-863.

7.	Riedl M, Banke IJ, Goronzy J, et al. Patients with Small Acetabular Cartilage Defects Caused by Femoroacetabular Impingement Do Not Benefit from Microfracture. *J Clin Med*. 2022;11(21):6283.

8.	Riedl M, Lenz JE, Goronzy J, et al. Save the subchondral bone plate: Debridement versus bone marrow stimulation in acetabular cartilage defects over 60 months of follow-up. *Knee Surg Sports Traumatol Arthrosc*. 2024;32(9):2395-2405.

9.	Su T, Ao Y, Yang L, Chen GX. Tissue Regrowth and Its Vascularization Through Bone Marrow Stimulation: Microfracture at the Acetabular Rim for Irreparable Labral Tear in a Porcine Model. *Am J Sports Med*. 2023;51(4):1024-1032.

10.	Wang KC, Frank RM, Cotter EJ, et al. Long-term Clinical Outcomes After Microfracture of the Glenohumeral Joint: Average 10-Year Follow-up. *Am J Sports Med*. 2018;46(4):786-794.
